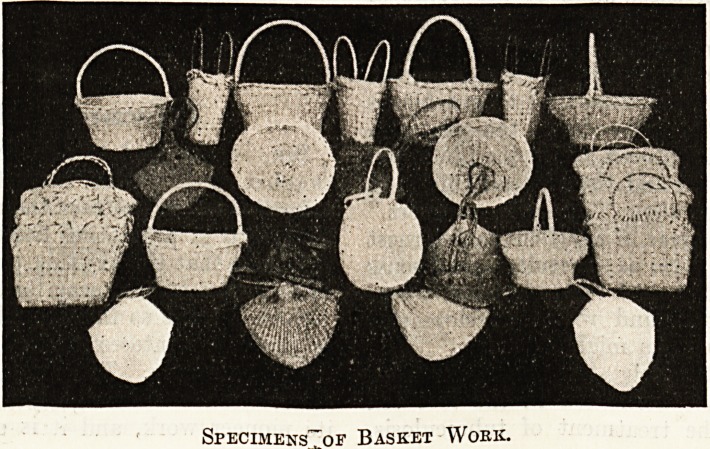# The High Grade Mental Defective

**Published:** 1923-11

**Authors:** 


### THE HIGH-GRADE MENTAL DEFECTIVE.

A PARTICULARLY useful work for the educable mentally defective boy is being done at the Besford Court Catholic Home, Worcestershire, under the administration of Monsignor Newsome. The home is intended for the high-grade "moron," who requires not only special teaching and training, but also preliminary, concurrent and continuous hospital treatment. For such a child, as the Annual Report points out, the ordinary elementary school education is of no value and the training in special day schools very little better. The teaching given for a few hours during the day at the best is restricted to a certain measure of success in imparting the knowledge of the "3 R's." This knowledge later may prove to be absolutely useless, and is utterly incapable of correcting the moral instability which is the dominant feature of such children's characters, and from which the whole danger to society and to themselves is liable to develop in after years.

Besford Court, North Side.

In the training of the high-grade "moron" money may be spent with the certainty of seeing value produced, as is shown by the satisfactory results achieved at Besford Court. A common mistake is to attach undue importance to subjects which are purely academic. It must be remembered that the mentally deficient child can never be taught to earn his living by his head; he must be taught to do so by his hands. Industrial and moral training are always given the premier place at Besford Court. It is not implied that a child is not instructed in the "3 R's" according to his capacity, but the time devoted to such academic matters is never allowed to encroach seriously upon that given to more important subjects. The more advanced pupils are finally taught some definite occupation. Mat-making, boot-finishing, carpentry, bookbinding, etc., are within the abilities of the high-grade defective. All the boys are taught to make their own beds and to black their own boots, while many make their own jerseys.

The Open Cloister.

The children learn to take pride in the neatness of their attire, in cleanliness of the hands, teeth, etc. They are encouraged to keep their clothes well brushed and to present a general "well-groomed" appearance. Nothing is more educational in awakening and fostering self-respect. It is very desirable that the boys should enter the home at an early age. The aim is that every boy should be capable, at 16 years of age, of going out into the world and, under suitable supervision, earning a portion, at least, of his livelihood. But it is clear that, if cases are not sent until the boys have reached the age of 12 or 13, sufficient training cannot be crowded into the remaining years for the boy to be in a fit state to leave the home at 16. The developments of the future include the provision of training for defective girls and the beginning, at least, of an industrial colony for lads up to the ages of 18 or 20.

Specimens of Basket Work.

### Silent Roads Outside Hospitals.

Sections of rubber roadway like that which it is proposed should be placed around the Cenotaph are being suggested in other places—for example, along the front of hospitals in busy towns. Hitherto such "floors" have been tried within hospitals only. The cost is the chief difficulty which we may expect to be solved in time.

## Figures and Tables

**Figure f1:**
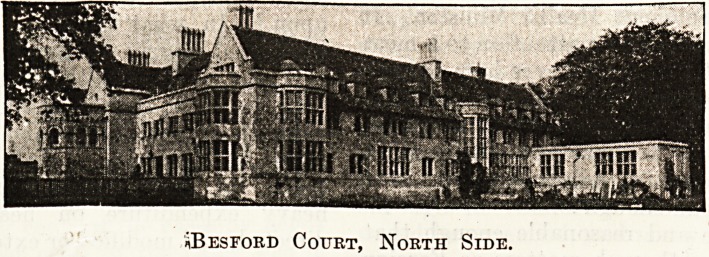


**Figure f2:**
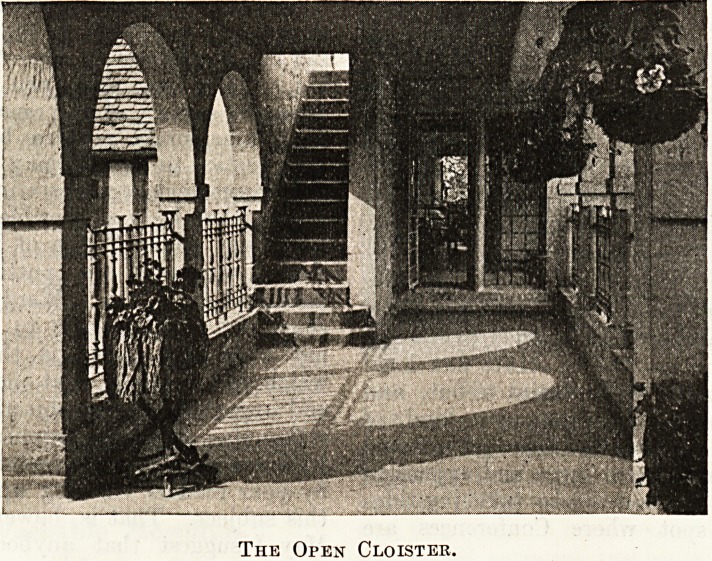


**Figure f3:**